# Diagnostic Challenge in a Patient with Severe Anion Gap Metabolic Acidosis

**DOI:** 10.1155/2015/272914

**Published:** 2015-05-31

**Authors:** Eugene M. Tan, Ejaaz Kalimullah, M. Rizwan Sohail, Kannan Ramar

**Affiliations:** ^1^Department of Internal Medicine, Mayo Clinic, 200 First Street SW, Rochester, MN 55905, USA; ^2^Department of Emergency Medicine, Loyola University Medical Center, 2160 S. 1st Avenue, Maywood, IL 60153, USA; ^3^Division of Infectious Diseases, Mayo Clinic, 200 First Street SW, Rochester, MN 55905, USA; ^4^Division of Pulmonary and Critical Care Medicine, Mayo Clinic, 200 First Street SW, Rochester, MN 55905, USA

## Abstract

The approach to the patient with acute renal failure and elevated anion and osmolal gap is difficult. Differential diagnoses include toxic alcohol ingestion, diabetic or starvation ketoacidosis, or 5-oxoproline acidosis. We present a 76-year-old female with type 2 diabetes mellitus, who was found at home in a confused state. Laboratory analysis revealed serum pH 6.84, bicarbonate 5.8 mmol/L, pCO2 29 mmHg, anion gap 22.2 mmol/L, osmolal gap 17.4 mOsm/kg, elevated beta-hydroxybutyrate (4.2 mmol/L), random blood sugar 213 mg/dL, creatinine 2.1 mg/dL, and potassium 7.5 mmol/L with no electrocardiogram (EKG) changes. Fomepizole and hemodialysis were initiated for presumed ethylene glycol or methanol ingestion. Drug screens returned negative for ethylene glycol, alcohols, and acetaminophen, but there were elevated urine levels of acetone (11 mg/dL). The acetaminophen level was negative, and 5-oxoproline was not analyzed. After 5 days in the intensive care unit (ICU), her mental status improved with supportive care. She was discharged to a nursing facility. Though a diagnosis was not established, our patient's presentation was likely due to starvation ketosis combined with chronic acetaminophen ingestion. Acetone ingestion is less likely. Overall, our case illustrates the importance of systematically approaching an elevated osmolal and anion gap metabolic acidosis.

## 1. Introduction

The presence of an osmolal gap in a patient with elevated anion gap metabolic acidosis typically alerts clinicians to toxic alcohol exposures, such as methanol, ethylene glycol, diethylene glycol, propylene glycol, or isopropanol. However, other disorders should be considered, such as diabetic or starvation ketoacidosis, acute kidney injury, chronic kidney disease, lactic acidosis, and salicylate intoxication, though not all of them create an osmolal gap [1]. However, to encompass an ever growing list of conditions resulting in elevated anion gap metabolic acidosis, particularly to account for 5-oxoproline (pyroglutamic acid) that is elevated in the setting of chronic acetaminophen ingestion, a new mnemonic was developed called GOLD MARK. This acronym stands for glycols, oxoproline (5-oxoproline also called pyroglutamic acid), L-lactate, D-lactate, methanol, aspirin, renal failure, and ketoacidosis [2]. We present a patient with an elevated anion gap metabolic acidosis and osmolal gap in the absence of toxic alcohol exposure, to illustrate the importance of using a systematic approach to arrive at the final diagnosis.

## 2. Case Presentation

A 76-year-old female with type 2 diabetes mellitus (hemoglobin A1c 6.3 on metformin therapy), overweight (BMI 28), and history of benzodiazepine and opiate use was found at home confused and lethargic, surrounded by water bottles containing pink fluid. A red-and-white pill with a #5 inscribed (likely acetaminophen/oxycodone) was next to her, along with dark brown emesis stains. She was taking high doses of acetaminophen (sometimes exceeding 8 Extra Strength tabs daily) for back and leg pain for the past year. She used to take hydrocodone/acetaminophen but was no longer being prescribed this medication. She had been depressed and was previously on sertraline but had self-discontinued the drug for unknown reasons. Although she was never formally diagnosed with dementia, her daughter mentioned she had difficulty taking care of herself and was not eating, drinking, and taking medications appropriately for several weeks at least. She became progressively somnolent and was admitted to the ICU.

**Figure 1:**
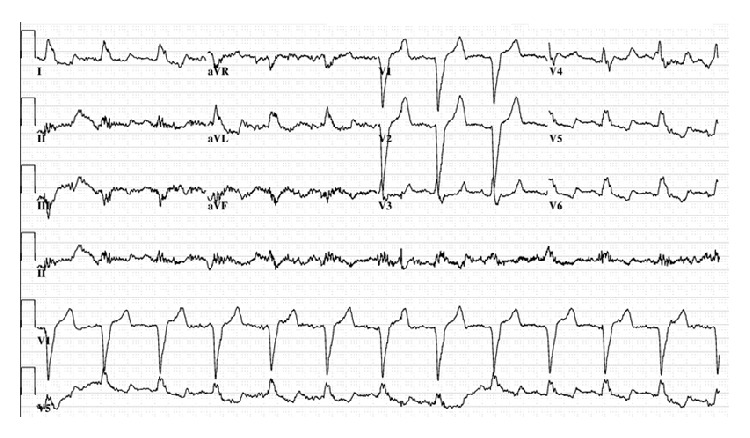
EKG showed prolonged QT, left bundle branch block, and left axis deviation but no T wave changes compared to a prior EKG.

On admission, venous blood gas revealed serum pH 6.84, serum bicarbonate 5.8 mmol/L, and anion gap 22.2 mmol/L (Na = 138 mmol/L, Cl = 110 mmol/L), consistent with an anion gap metabolic acidosis. Other laboratory values included BUN 52.6 mg/dL, creatinine = 2.1 mg/dL, elevated beta-hydroxybutyrate (4.2 mmol/L), normal lactate 1.5 mmol/L, and random blood sugar 213 mg/dL. See Table 1 for additional laboratory values. Serum osmolality was elevated at 324 mOsm/kg. As her calculated osmolality was 306.6 mOsm/kg (Na = 138 mmol/L, BUN = 52.6 mg/dL, and glucose = 213 mg/dL), there was an osmolal gap of 17.4 mOsm/kg. Her potassium was 7.5 mmol/L, but an EKG showed no peaked T waves (Figure 1). However, she was given intravenous bicarbonate, insulin, glucose, calcium, and nebulized albuterol to treat the hyperkalemia. Given the elevated anion and osmolal gap and suspicion for ethylene glycol or methanol ingestion, 2 doses of fomepizole (670 mg IV Q12H) and 2 hemodialysis sessions were initiated while drug levels of these alcohols were still pending.

Serum and urine drug screens returned negative for ethylene glycol, methanol, ethanol, isopropanol, acetaminophen, and salicylates, but there were elevated levels of acetone (11 mg/dL) in the urine. Serum acetone was however not detectable. Her daughter later reported seeing a bottle of nail polish remover in her mother's house, though a direct ingestion history was not available. Due to the elevated beta-hydroxybutyrate and acetone levels, starvation ketosis was also entertained, and the patient was given thiamine along with intravenous glucose followed by enteral nutrition with close monitoring for refeeding syndrome. After a 5-day stay in the ICU, her mental status improved with supportive care. She was unable to clearly recount what occurred prior to her hospital admission, however. Creatinine returned to baseline of 0.8 mg/dL. She was transferred to the general floor and discharged to a nursing facility.

## 3. Discussion

Our case illustrates the diagnostic challenges in establishing an etiology for our patient's elevated anion gap metabolic acidosis and osmolal gap in the absence of alcohols such as methanol and ethylene glycol and in the presence of elevated beta-hydroxybutyrate and acetone levels. Despite the finding of high urine acetone concentrations, this may not necessarily mean that our patient ingested acetone as acetone may not typically produce laboratory findings of high anion gap and osmolal gap. However, acetone can possibly yield an elevated anion gap via ketosis or kidney injury with subsequent uremia, which our patient did have. The acetone metabolites, acetol and 1,2-propanediol, can possibly cause an increased osmolal gap as well [3].

In the literature, there are two case reports from Sweden in which patients admitted drinking alcohol but had only high concentrations of acetone in urine (0.10 g/dL and 0.052 g/dL). No ethanol or isopropanol was found. The investigators surmised that this was due to the longer elimination half-life of acetone (17–27 hours) versus isopropanol (1–3 hours). High concentrations of acetone in body fluids are found with ingestion of isopropanol because isopropanol is converted into acetone by oxidation with class I isoenzymes of hepatic alcohol dehydrogenase. Acetone is mostly excreted unchanged in the breath and urine, but some may be oxidized by cytochrome P450 enzymes via slow detoxification [4].

Abnormally high concentrations of acetone may also be found in the blood, breath, or urine of patients with diabetic or starvation ketoacidosis. When carbohydrate reserves are depleted, the body breaks down fat to produce ketone bodies such as beta-hydroxybutyrate, acetoacetate, and acetone. Acetone may be involved in the conversion of fat to carbohydrate during prolonged periods of fasting [4]. Our patient did have type 2 diabetes mellitus, and she had an elevated beta-hydroxybutyrate of 4.2 mmol/L, but her random blood sugar was 213 mg/dL, which did not meet the criteria for diabetic ketoacidosis. Her metformin use can cause lactic acidosis via blockade of gluconeogenesis, glycogenolysis, mitochondrial respiration, and oxidative phosphorylation, but her lactate was normal [5]. Metformin is unlikely to cause ketoacidosis, and no literature has been found. Hence, the elevated acetone and beta-hydroxybutyrate levels probably suggest starvation ketoacidosis, which was later confirmed by the patient and her daughter and based on her malnourished state.

Though less likely in our patient, we have to also be vigilant about other alcohol ingestions such as diethylene or propylene glycol as these alcohols may not be detected by laboratory testing. It is also worth noting that our patient had a history of chronic acetaminophen ingestion for pain, which is associated with reduced plasma glutathione levels, therapeutic or low acetaminophen levels, and elevation of 5-oxoproline levels in serum and urine. The exact mechanism for 5-oxoproline production is unknown, but the incidence of severe anion gap metabolic acidosis from 5-oxoproline and chronic acetaminophen ingestion is higher if the patient has comorbidities such as malnourishment, pregnancy, vegetarian diet, sepsis, chronic renal insufficiency, or hepatic dysfunction. Diagnosis is confirmed by measuring urine 5-oxoproline, acetoacetate, 3-hydroxybutyrate, lactate, and 2-hydroxy acids [2]. The initial acetaminophen level in our patient was negative. Though our patient did not have a 5-oxoproline measurement, based on her history of chronic acetaminophen ingestion, decreased oral intake, and negative tests for alcohols, we speculate that her 5-oxoproline measurement might have been elevated.

In conclusion, we strongly suspect that our patient's abnormal laboratory values were likely to be explained by starvation ketosis in the setting of malnourishment and chronic acetaminophen ingestion. Though possible, it is less likely that our patient ingested acetone. Our case illustrates the importance of systematically approaching a diagnostic dilemma of a patient with elevated anion gap metabolic acidosis and an elevated osmolal gap, in the absence of toxic alcohols, to arrive at a diagnosis.

## Figures and Tables

**Table 1:** Admission laboratory values.

Laboratory	Value	Reference
Hemoglobin	11.5 g/dL	12.0–15.5 g/dL
Hematocrit	35.2%	34.9–44.5%
Leukocytes	26.6 × 10^3^ cells/uL	3.4–10.5 × 10^3^ cells/uL
Leukocyte differential	85.6% neutrophils, 7.5% lymphocytes, 6.8% monocytes, 0% eosinophils, and 0.1% basophils	41–77% neutrophils, 20–45% lymphocytes, 0–12% monocytes, 0–6% eosinophils, and 0–2% basophils
Platelets	298 × 10^3^ cells/uL	150–450 × 10^3^ cells/uL
INR	1.3	0.8–1.2
Prothrombin time	17.0 seconds	9.5–13.8 seconds
Sodium	138 mM/L	136–148 mM/L
Potassium	7.5 mM/L	3.5–5.0 mM/L
Creatinine	2.1 mg/dL	0.6–1.1 mg/dL
Blood urea nitrogen	52.6 mg/dL	6.0–21.0 mg/dL
Chloride	111 mmol/L	100–108 mmol/L
Bicarbonate	5.8 mmol/L	21–32 mmol/L
Calcium	8.8 mg/dL	8.4–10.4 mg/dL
Total protein	7.4 g/dL	6.3–8.2 g/dL
Magnesium	2.3 mg/dL	1.6–2.3 mg/dL
Alkaline phosphatase	83 IU/L	55–142 IU/L
AST	41 IU/L	15–46 IU/L
ALT	41 IU/L	10–43 IU/L
Total bilirubin	<0.40 mg/dL	0.10–1.00 mg/dL
Blood and urine cultures	No growth	No growth
